# Methodological Aspects of ELISA Analysis of Thioredoxin 1 in Human Plasma and Cerebrospinal Fluid

**DOI:** 10.1371/journal.pone.0103554

**Published:** 2014-07-30

**Authors:** Mathias Lundberg, Sophie Curbo, Kathrin Reiser, Thomas Masterman, Sten Braesch-Andersen, Irene Areström, Niklas Ahlborg

**Affiliations:** 1 Department of Clinical Science and Education, Karolinska Institutet, Stockholm, Sweden; 2 Department of Laboratory Medicine, Karolinska Institutet, Huddinge, Sweden; 3 Department of Clinical Neuroscience, Karolinska Institutet, Stockholm, Sweden; 4 Mabtech, Nacka, Sweden; 5 Department of Immunology, Stockholm University, Stockholm, Sweden; Innsbruck Medical University, Austria

## Abstract

Thioredoxin-1 (Trx1) is a protein antioxidant involved in major cellular processes. Increased plasma levels of Trx1 have been associated with human diseases suggesting that Trx1 is a marker for oxidative stress with putative clinical use. However, the reported mean levels of Trx1 in the control cohorts vary a hundred-fold between studies (0.8–87 ng/ml), possibly due to methodological differences between the capture ELISA used in the different studies. The aim of this study was to investigate methodological aspects related to the ELISA measurement of Trx1. ELISAs utilizing different capture and detection combinations of antibodies to Trx1 and as well as recombinant human (rh) Trx1 standards from two sources were characterized. The different ELISAs were subsequently used to measure Trx1 in human plasma and cerebrospinal fluid samples (CSF) from healthy donors and from patients with various neurological diagnoses. The Trx1 standards differed in their content of monomeric and oligomeric Trx1, which affected the ELISAs composed of different antibody combinations. Thus, the levels of Trx1 determined in human plasma and CSF samples varied depending on the antibody used in the ELISAs and on the rhTrx1 standard. Furthermore, the relevance of preventing interference by heterophilic antibodies (HA) in human plasma and CSF was investigated. The addition of a HA blocking buffer to human samples drastically reduced the ELISA signals in many samples showing that HA are likely to cause false positive results unless they are blocked. In conclusion, the study shows that the design of a Trx1 ELISA in regards to antibodies and standards used has an impact on the measured Trx1 levels. Importantly, analyses of human plasma and CSF without preventing HA interference may obscure the obtained data. Overall, the results of this study are crucial for the improvement of future studies on the association of Trx1 levels with various diseases.

## Introduction

Thioredoxin-1 (Trx1) is a 12 kDa ubiquitous oxido-reductase protein and is despite its small size a biochemically complex protein that catalyses protein disulfide reductions [1]. The active site of Trx1 comprises of two thiols (Cys32-Gly-Pro-Cys35) that form a disulfide upon oxidation. Trx1 has three additional structural cysteine residues outside its active site that may form intra- and/or intermolecular disulfide bonds. The extent and pairing of the structural cysteines in the oxidized form of Trx1 appear to be related to the oxidizing agent [2]. Trx1 is kept in a reduced state by thioredoxin reductase-1 and ultimately NADPH [3], but not all redox forms can be reduced by thioredoxin reductase-1. Some variants can only be reduced by a strong reducing agent such as dithiothreitol (DTT) [2]. The bioactivity of Trx1 may also be regulated by glutathionylation and nitrozylation [4], [5]. Trx1 plays a major role in keeping the intracellular milieu in a reduced state [3] and has a specific activity in regulating the bioactivity of numerous proteins including apoptosis factors, transcription factors and receptors [5]. Moreover, it is involved in the defence against oxidative stress and its expression is strongly induced in response to it [6], [7].

Several studies have shown an association between increased plasma levels of Trx1 and a broad spectrum of severe somatic and psychiatric diseases, which indicate a state of oxidative stress in these patients [7], [8], [9], [10], [11], [12], [13], [14], [15]. This suggests that Trx1 may be used as a state and/or trait biomarker of potential clinical use. Despite the apparent correlation between plasma Trx1 levels, oxidative stress and disease state in these studies, the measured concentration of Trx1 varies considerably amongst the control groups; the mean plasma concentration of Trx1 ranges from 0.8 ng/ml to 87 ng/ml. The discrepancy between these studies suggests that the applied capture ELISAs differ in their measurement of Trx1. This has important implications when evaluating results and limits the possibility of comparing studies.

Previous analyses of Trx1 in human samples have been conducted with both non-commercial and commercial capture ELISAs based on a variety of antibody combinations and Trx1 standards [8], [9], [10], [11], [12], [13], [15], [16], [17], which could have influenced the quantitation. In addition, the measurement can also be affected by interference from heterophilic antibodies (HA) that bind to immunoglobulins from goat, mouse, rat and other mammals and thereby cross-link the capture and detection antibodies used [18], [19]. HA occur in 30–70% of all humans and are found in plasma, serum and cerebrospinal fluid (CSF) [20]. The HA activity may be limited by sample dilution and/or by using sample dilution buffer containing an excess of animal antibodies blocking the HA from cross-linking the assay reagent [21]. Several studies have shown that HA interference can cause entire studies to be built on false positive capture immunoassay results [18], [19], [21], [22].

The present study was undertaken to investigate relevant methodological aspects for the measurement of Trx1 by capture ELISA including evaluation of the impact of the antibodies used, the redox status of Trx1 ELISA standards and the blocking buffer for HA.

## Materials and Methods

### Antibodies and recombinant proteins

Antibodies used for the ELISA as well as for Western blotting were the Protein G-purified mouse monoclonal antibody (mAb) 2G11 (Mabtech, Nacka Strand, Sweden), [23] and a goat polyclonal antibody (pAb) to human Trx1 purified on human Trx1 (IMCO, Stockholm, Sweden). Antibodies were biotinylated using a 20X excess of EZ-link Sulfo N-hydroxysuccinimide ester biotin (Pierce, Rockford, IL, USA) according to the manufacturer’s instructions followed by buffer exchange over a NAP5 column (Amersham Biosciences, Uppsala, Sweden) to phosphate-buffered saline (PBS) with 0.09% sodium azide to remove excess free biotin. Full-length recombinant human (rh)Trx1 produced in *Escherichia coli* was obtained from IMCO and from R&D Systems (Abingdon, UK). Notably, according to the manufacturers, the Trx1 from R&D was treated with DTT prior to lyophilization whereas the Trx1 from IMCO was not.

### DTT reduction of recombinant human Trx1

100 µg/ml rhTrx1 (8.6 µM) dissolved in PBS was incubated with a 5-fold molar excess of DTT (Sigma-Aldrich, St. Louis, MO, USA) and 5 mM ethylenediaminetetraacetic acid (EDTA; Sigma-Aldrich) for 30 min at 37°C in a water bath. When analysing the DTT-treated Trx1 in ELISA, the concentration of DTT was below 50 nM at the highest concentration of Trx1 tested. 5 mM EDTA was added to all ELISA buffers when analysing DTT-treated Trx1; the inclusion of EDTA in the buffers did not have any impact on the detection of Trx1 not treated with DTT.

### SDS-PAGE and Western blot

rhTrx1 (DTT-treated or non-treated) was mixed with NuPage LDS sample buffer (Invitrogen, Carlsbad, CA, USA), kept at 70°C for 10 min and resolved under non-reducing conditions on NuPAGE 4–12% gradient Bis-Tris gels (Invitrogen) in a XCELL II Electrophoresis cell (Novex, San Diego, CA, USA) using NuPage MOPS running buffer (Invitrogen). A pre-stained standard (SeeBlue Plus 2; Invitrogen) was included as reference. For SDS-PAGE analysis, 5 µg Trx1 was loaded per lane and the gel was subsequently stained with Simply Blue Safestain (Invitrogen). For Western blot analysis, 1 µg Trx1 was loaded per lane and the proteins were transferred to 0.2 µm pore size nitrocellulose membrane (Invitrogen) after separation using a MiniTrans-Blot apparatus (Bio-Rad, Hercules, CA, USA) with 20 mM Tris pH 8.6. Membranes were blocked for 1 h at room temperature with 4% fetal calf serum (FCS) in PBS. After washing with PBS, the membranes were incubated with 1 µg/ml of biotinylated mAb 2G11 or pAb in PBS with 0.5% FCS. The membranes were washed and incubated for 1 h at room temperature with streptavidin-alkaline phosphatase (Mabtech) diluted 1∶1000 in PBS, washed again and developed with BCIP/NBT Plus (Mabtech) for 5 min before being rinsed in tap water.

### Analysis of the recognition of rhTrx1 by different antibody combinations in ELISA

The recognition of rhTrx1 from two different sources was evaluated using different combinations of mAb 2G11 or pAb as capture and biotinylated detection reagents. Capture antibodies were diluted in PBS (pH 7.4) and absorbed to 96-well Maxisorp plates (Nunc, Roskilde, Denmark) overnight at 4°C. All of the steps below were carried out at room temperature and all incubations volumes were 100 µl/well. Wells were washed 5 times between incubations with 200 µl of PBS containing 0.1% Tween 20. After coating, the wells were blocked for 1 h with incubation buffer (PBS containing 0.05% Tween 20 and 1% bovine serum albumin) and duplicates of rhTrx1 at consecutive two-fold dilutions ranging from 50 to 0.01 ng/ml were added and incubated for 2 h. Afterwards, biotinylated detection antibody and subsequently streptavidin-horseradish peroxidase conjugate (SA-HRP) (50 ng/ml; Mabtech) were incubated for 1 h each. rhTrx, detection antibody and SA-HRP were diluted in incubation buffer. The assay was developed by addition of the ready-to-use substrate TMB (Mabtech) and stopped after 15 min using 1 M H_2_SO_4_. Absorbance at 450 nm (with absorbance at 650 nm subtracted) was determined using an ELISA reader (Labsystems, Helsinki, Finland). In pilot tests, optimal signal-to-noise ratios were established by cross-checking different concentrations of capture and detection antibodies. The final concentrations of antibodies used in the different capture/detection combinations were pAb/pAb-biotin (1.0/0.1 µg/ml), mAb/pAb-biotin (1.0/0.2 µg/ml) and mAb/mAb (2.0/0.5 µg/ml). ELISA sensitivity was calculated for each run of a plate assay by calculating two times the standard deviation for the blank + the OD for the blank (analysis with no sample or standard). All OD read outs below this value was set as “non detectable”.

### Analysis of Trx1 in plasma samples

Plasma from healthy subjects (n = 18) was prepared from venous blood obtained in EDTA Vacutainer vials (Becton Dickinson, Franklin Lakes, NJ, USA) by two centrifugations at 1,000×g (10 min). Blood was obtained with informed consent. Within one hour after collection all samples were stored in aliquots at −20°C until tested. All plasma samples were analyzed within 6 months. When used for analysis by ELISA, plasma was either diluted in incubation buffer (as above) or HA blocking buffer designed to prevent HA activity. The ELISA was performed as described above. To further assess the potential problem with HA, two selected samples were also analysed by ELISA variants where the capture antibody had been replaced with a capture antibody with irrelevant specificity i.e. antibodies not matching the Trx1-specific detection antibody. MAb 2G11 was hence replaced with an irrelevant mouse mAb and the pAb to Trx1 with and irrelevant goat pAb.

### Analysis of Trx1 in cerebrospinal fluid

Cerebrospinal fluid (CSF) samples were collected from patients (n = 57) at the Division of Neurology and the Division of Geriatrics at Huddinge University Hospital as previously described for routine diagnostics and stored at –80°C [24], [25]. The patient population consisted of 30 females and 27 males with a mean age of 50 years. The patient population had the following diagnoses: control patients (n = 8), multiple sclerosis (n = 20), demyelinating polyneuropathies (n = 10), Alzheimer’s disease/dementia (n = 9), and cognitive impairment (n = 10). The control patient group consisted of patients with headache of uncertain background without any clinical or laboratory signs of CNS-diseases. The patients had a normal CSF/serum albumin ratio [26], [27] and an absence of blood cells in the CSF. For the analysis, CSF, standard (rhTrx1) and the detection antibody were diluted in incubation buffer or the HA blocking buffer (Mabtech) to evaluate the role of HA. The ELISA was performed as described above.

### Statistical analysis

Bland-Altman assessment for agreement was used to compare the impact of incubation buffer or HA blocking buffer for the two ELISA formats. The absolute difference in ELISA measurements was correlated to the mean of both measurements for each subject when using the same buffer system. Correlations between the ELISA formats in HA blocking buffer or incubation buffer, respectively, were determined with Spearman’s rank. Trx1 levels determined in plasma or CSF are presented as median and interquartile range (25^th^–75th percentiles). Comparisons were made with Wilcoxon signed rank test. All calculations were done with XLSTAT software.

### Ethical statement

All investigations on patients with neurological or geriatric diseases were approved by the ethics committee of Karolinska University Hospital (approval nos. 488/98, 303/99 and 274/01). Samples were collected by physician-performed lumbar puncture and stored afterwards at −80°C as part of the planned diagnostic workup. With regard to the use of the samples for research purposes, the guidelines of the ethics committee of Karolinska University Hospital required neither written nor informed consent from participating subjects. Subjects were informed, however, that anonymized samples would be stored for potential later use, including research, and given the opportunity to decline participation.

## Results

### SDS-PAGE and Western blot analysis of recombinant human Trx1

**Figure 1:**
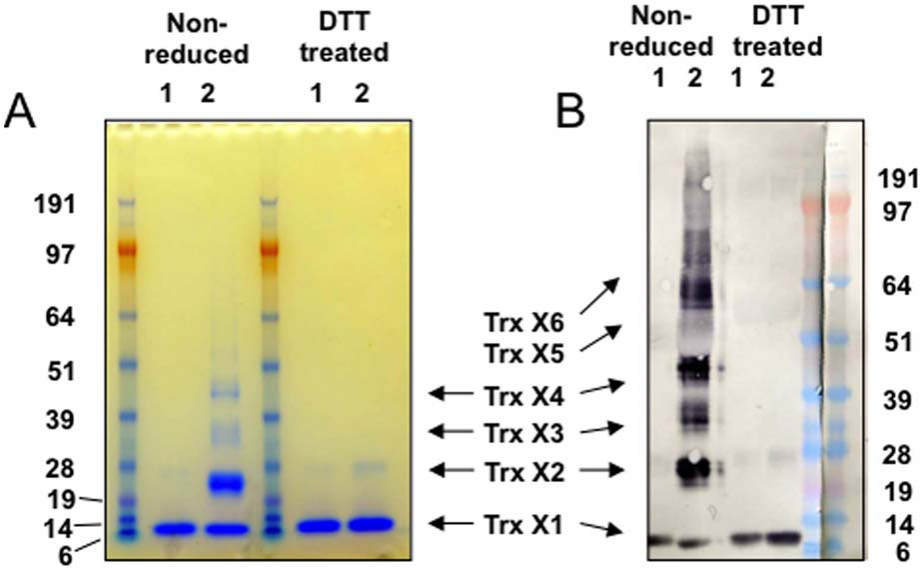
SDS-PAGE and Western blot analysis of recombinant human Trx1. A) SDS-PAGE analysis: Lanes were loaded with 5 µg of Trx1 from R&D (1) or Trx1 from IMCO (2). Trx1 samples to the right had first been treated with DTT for 30 min. The samples were separated under non-reducing conditions on the gel. B) Western blot analysis: Lanes were loaded with either 1 µg of Trx1 from R&D (1) or Trx1 from IMCO (2), treated or not with DTT as above, After separation in SDS-PAGE as above and transfer to nitrocellulose membrane, Trx1 was detected by mAb 2G11. The experiments were repeated with consistent results.

It was hypothesised that commercially available rhTrx1 differ in their content of various redox forms of Trx1 due to differences in preparation and/or storage conditions, which can be of importance when rhTrx1 is used as an ELISA standard. Therefore, rhTrx1 from two sources were analysed by non-reducing SDS-PAGE to determine their content of various redox forms. The results show that rhTrx1 from IMCO contained monomeric, dimeric and oligomeric forms of rhTrx1, which will be referred to as oxidised rhTrx1 (Fig. 1A). In contrast, rhTrx1 from R&D consisted predominantly of monomers and no detectable oligomers, which will be referred to as reduced rhTrx1 (Fig. 1A). Treatment of rhTrx1 from IMCO using DTT yielded predominantly monomers.

To verify that the bands in the SDS-PAGE corresponded to different forms of Trx1, a Western blot was performed using mAb 2G11 (Fig. 1B). The analysis confirmed the presence of different redox forms in the rhTrx1 from IMCO, as seen in SDS-PAGE (Fig. 1A). Due to the higher detection sensitivity of the Western blot, also larger multimers than observed in SDS-PAGE were identified.

### Recognition of rhTrx1 in different redox states by ELISAs based on different antibodies

**Figure 2:**
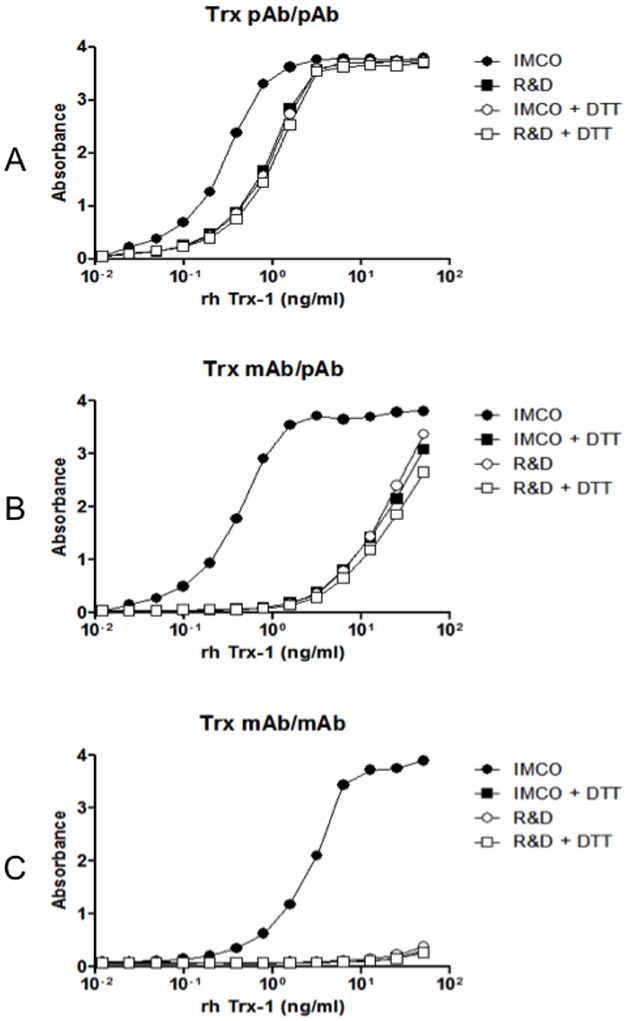
ELISA analysis of two different recombinant Trx1 by capture ELISA. Antibody combinations used for capture/detection were pAb/pAb (A) and mAb/pAb (B) and mAb/mAb (C). Trx1 standards were from IMCO and R&D. Both Trx1 standards were also analysed after being reduced by DTT. Data shown is from one of three experiments with consistent results.

Quantification of Trx1 in human samples by ELISA is dependent on the reaction of the antibodies used as capture and detection reagents with the rhTrx1 used as standard *versus* their reaction with native Trx1 in samples. To investigate the recognition of different Trx1 redox forms, two antibodies previously used to measure Trx1 [16], [28], [29], goat pAb and mAb 2G11, were used in different capture and detection combinations in an ELISA. Oxidized rhTrx1 as well as reduced rhTrx1 were used as standards. Both standards were also analysed after being treated with DTT. The usage of pAb for capture and pAb-biotin for detection (Fig. 2A) as well as the usage of mAb 2G11 for capture together with pAb-biotin for detection (Fig. 2B) resulted in better detection of the oxidized Trx1 compared to the reduced Trx1. However, reversing the ELISA system using pAb for capture and mAb 2G11 for detection resulted in a low detection of all Trx1 variants, hence this combination of antibodies was not further investigated (data not shown). Finally, using the mAb both for capture and detection resulted in recognition restricted to oxidized Trx1 (Fig. 2C).

### Validation of ELISA analysis of human plasma samples

Having shown that two previously reported capture ELISAs based on the combinations of capture and detection antibodies had different specificity to monomeric (reduced) and polymeric (oxidized) Trx1, the ELISAs recognition of plasma Trx1 was compared. However, analysis of plasma proteins by capture ELISA has been shown to be obscured by the possible interference by HA commonly occurring in plasma [18], [19], [21], [22]. To investigate the influence of HA, two plasma samples known to display high and low HA activity, respectively, were selected for analysis using pAb/pAb and mAb/pAb Trx1 ELISA and were diluted in either incubation buffer or in HA blocking buffer. To confirm the presence of HA, the capture pAb and the capture mAb in the ELISAs were also replaced with irrelevant capture pAb and mAb, respectively; any signal obtained with a capture antibody with irrelevant specificity (i.e. not reactive with Trx1) can be considered as a false positive signal.

**Figure 3:**
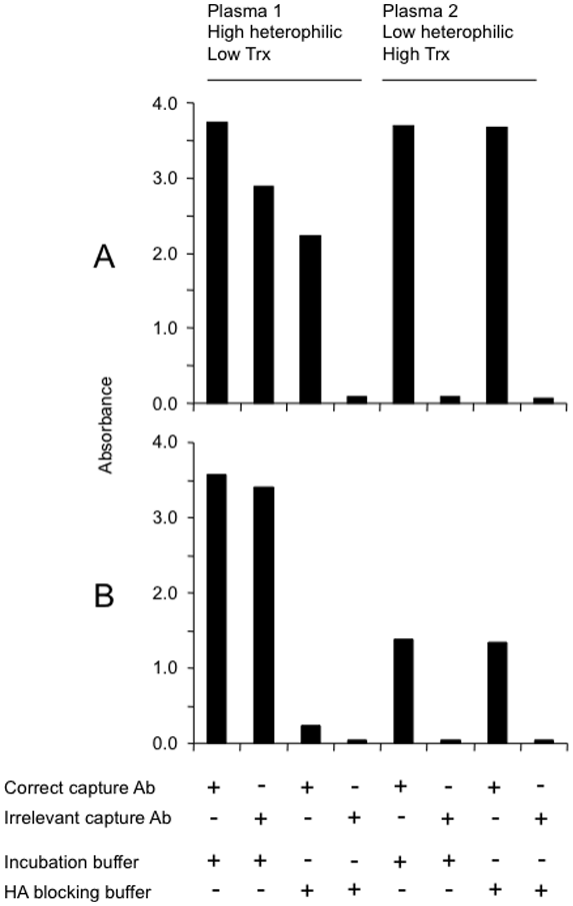
Heterophilic antibody (HA) interference in the different capture ELISA setups. Impact of HA on the detection of Trx1 in two human plasma samples. Two plasmas were diluted 5-fold in incubation buffer or HA blocking buffer and analyzed by Trx1 ELISA. A: analysis with pAb/pAb system where the capture pAb also was replaced by an irrelevant goat pAb. B: analysis with mAb/pAb system where the capture mAb also was replaced by an irrelevant mouse mAb. The experiment was repeated with similar results.

As shown in Figure 3, plasma sample 1 yielded high absorbance when diluted in incubation buffer and analysed by the ELISA using both specific and irrelevant capture antibodies. After diluting the sample in HA blocking buffer, the absorbance obtained with irrelevant capture antibodies was lost whereas the ELISAs with specific capture antibodies still yielded a signal, albeit low; the results indicate high levels of HA and low, but detectable, levels of Trx1. In contrast, sample 2 diluted in either buffer showed low reactivity in the ELISAs using irrelevant antibodies whereas the use of correct capture antibodies yielded a high signal; results indicating low levels of HA but high levels of Trx1. Taken together, these ELISAs are strongly influenced by the presence of HA and accurate measurement of Trx1, in all samples, requires prevention of HA interference.

### Quantification of Trx1 in human plasma samples

**Figure 4:**
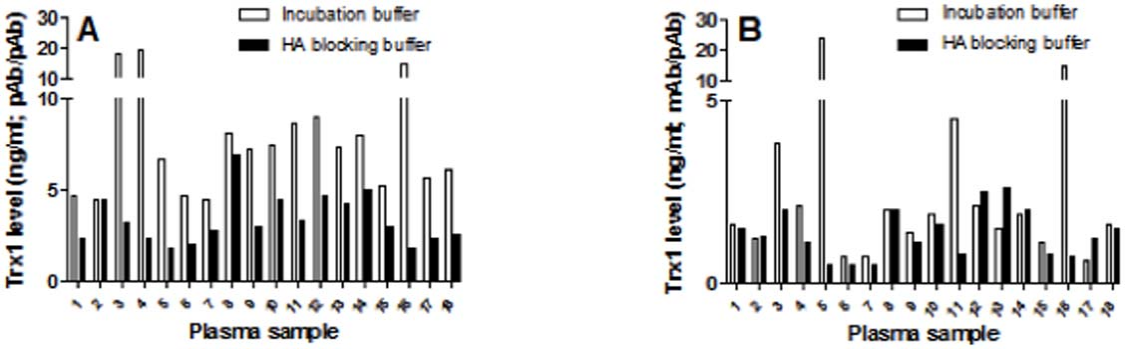
Analysis of the level of Trx1 in plasma samples by capture ELISA. Eighteen plasma samples from healthy blood donors were analysed for the level of Trx1 using the ELISA formats, pAb/pAb (A) and mAb/pAb (B), in the presence of either incubation buffer or a HA blocking buffer. One of two experiments with similar results is shown.

To further investigate the impact of different antibodies, rhTrx1 standards and HA interference on quantification of Trx1, Trx1 was measured in plasma samples from eighteen healthy donors. The ELISAs displaying reactivity with both monomeric and oligomeric Trx1 (pAb/pAb and mAb/pAb) could detect Trx1 in human plasma (Fig. 4) whereas the mAb/mAb-biotin ELISA, which is restricted to oligomeric Trx-1, did not yield detectable signals (data not shown).

The use of a HA blocking buffer had an important impact in both ELISAs. In the pAb/pAb system the blocking buffer reduced the detected levels of Trx1 2-fold in many samples compared to not using the HA blocking buffer. However, in some samples, the detected levels were reduced approximately 5-fold. In the mAb/pAb system, the HA blocking buffer did not affect the absorbance in many samples. However, in certain samples the measured levels were reduced more than 25-fold when using the HA blocking buffer (data not shown).

**Figure 5:**
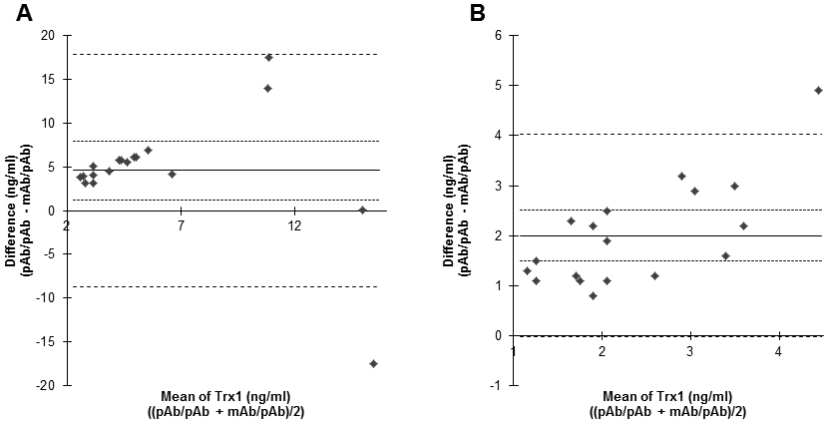
Agreement of the level of Trx1 in plasma samples by pAb/pAb and mAb/pAb capture ELISA. The agreement between the two ELISAs used for analysis of eighteen plasma samples from healthy blood donors and diluted in incubation buffer (A) or HA blocking buffer (B) was assessed with Bland-Altman analysis. One of two experiments with similar results is shown. ––– Bias •••••••••••• CI Bias (95%) – – – – CI (95%).

Notably, some samples displayed similar HA reactivity in the two ELISAs, whereas other samples displayed different degrees of HA activity. Bland-Altman analysis was used to study the agreement between the two ELISAs in the presence of the different buffers. In the presence of incubation buffer the results of the ELISAs resulted in a Bland-Altman plot with a bias of 4.572 (95% confidence interval, −8.727 to 17.872), while blocking HA resulted in a Bland-Altman plot with a lower bias of 2 (95% confidence interval, −0.025 to 4.025) (Fig. 5). The confidence interval for the difference in ELISA measurements in the presence of incubation buffer was markedly wider compared to the confidence interval for the difference in the Trx1 levels acquired in the presence of HA buffer. The median levels were also lower in the presence of HA buffer compared to incubation buffer for both types of ELISA. The median Trx1 level for the mAb/pAb ELISA in the presence of HA blocking buffer was 1.3 ng/ml (interquartile range 0.8–1.9 ng/ml) and slightly higher in the presence of incubation buffer, (1.8 ng/ml; interquartile range 1.2–2.1 ng/ml). The median level of Trx1 was significantly lower in the pAb/pAb ELISA in the presence of HA buffer (3 ng/ml; interquartile range 2.3–4.4) compared to when HA was not blocked 7.3 ng/ml (interquartile range 5.3–8.6 ng/ml) (p<0.01). The data demonstrate that the two assays quantify Trx1 in plasma in a similar manner, provided measures are taken to prevent HA interference.

Quantifications of Trx1 levels were made against a standard curve with monomeric Trx1. An additional quantification against oligomeric Trx1 standard yielded lower levels of Trx1 in samples due to the fact that both ELISA systems react more strongly with the oligomeric standard (data not shown).

### Quantification of Trx1 in human cerebrospinal fluid

To our knowledge there is only one previous study in which Trx1 levels have been analysed in CSF, and that study indicated that CSF may contain Trx1 [30]. The level of Trx1 was analyzed in CSF from patients with various neurological symptoms including patients with headache (without clinical or laboratory signs of CNS-disease), multiple sclerosis, demyelinating polyneuropathies, Alzheimer’s disease/dementia, and cognitive impairment using pAb/pAb and mAb/pAb Trx1 ELISA, respectively. The median level of Trx1 with the pAb/pAb ELISA was 0.80 ng/ml (interquartile range 0.36–1.84 ng/ml) and with the mAb/pAb ELISA 0.019 ng/ml (interquartile range 0.001–0.074 ng/ml). Notably, fourteen out of 57 samples analyzed with the mAb/pAb ELISA, were below the detection level. No significant difference was found between the patient groups (data not shown), possibly due to the low sample numbers in each patient population.

**Figure 6:**
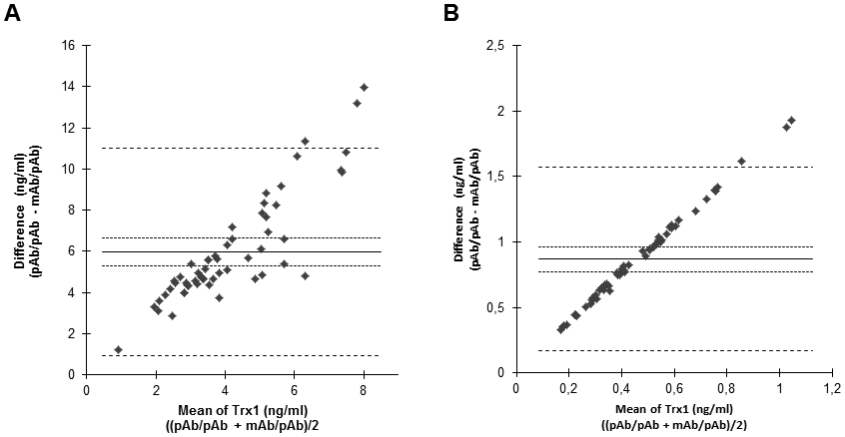
Agreement of the level of Trx1 in CSF samples by pAb/pAb and mAb/pAb capture ELISA. Fifty-seven CSF samples from patients with diverse diagnosis were analysed for the level of Trx1 using the ELISA formats pAb/pAb and mAb/pAb in the presence of either incubation buffer or a HA blocking buffer. The agreement between the two ELISA formats used for analysis of samples diluted in incubation buffer (A) or HA blocking buffer (B) was assessed with Bland-Altman analysis. One of two experiments with similar results is shown. ––– Bias •••••••••••• CI Bias (95%) – – – – CI (95%).

The analysis of CSF by the two ELISA formats described above were conducted using the HA blocking buffer to exclude a possible influence by HA. To study the agreement between the two ELISAs a Bland-Altman plot was established and showed to exhibit a bias of 0.87 (95% confidence interval, 0.17 to 1.57) (Fig. 6B). However, CSF has also been shown to contain HA with activity that interferes with the analysis of biological markers [22]. We thus decided to analyze the importance of preventing HA interference for the quantification of Trx1 in CSF. All CSF samples were diluted in incubation buffer (not blocking HA) and subsequently re-analyzed with the two ELISAs. The median level of Trx1 with the pAb/pAb ELISA was 6.3 ng/ml (interquartile range 3.6–13.8 ng/ml) and with the mAb/pAb ELISA 0.93 ng/ml (interquartile range 0.30–2.86 ng/ml). The Bland-Altman plot exhibited a higher bias of 5.98 (95% confidence interval, 0.93 to 11.03) (Fig. 6A). Studying the Bland-Altman plots it is evident that both plots exhibit a measurement error proportional to the mean. In these plots it means that the standard deviation increases with increasing mean values since the values of the mAb/pAb ELISA in some CSF samples were 100-folds lower than the values of the pAb/pAb ELISA and that some of the lower concentrations of Trx1 in the CSF samples were below detection limit in the mAb/pAb ELISA in the presence of HA blocking buffer. Comparison of the two Bland-Altman plots shows that the bias is markedly higher in incubation buffer when HA has not been blocked and it is also clear that the median range of the values is much higher in the absence of HA blocking buffer. Statistically the median levels are significantly higher for both ELISAs when HA have not been blocked (p<0.01). The correlations between the ELISAs determined with Spearman’s rank show that the correlation is better between the two ELISAs for CSF samples diluted in the HA blocking buffer (r_s_ = 0.66) than in incubation buffer (r_s_ = 0.37).

Altogether the data show that HA can affect samples differently in the two ELISA variants and that the presence of HA is a confounding factor in determination of Trx1 levels both in plasma and CSF.

## Discussion

In recent years a large number of studies have reported increased plasma Trx1 levels in various somatic and mental diseases indicating a state of oxidative stress [8], [9], [10], [11], [12], [13], [15], [16], [28]. These observations suggest that Trx1 may be used as state and/or trait marker with possible clinical use. However, the level of plasma Trx1 reported from these studies vary considerably probably due to methodological differences, which can have implications when analysing data or when comparing observations from different studies. The current study was conducted with the aim to determine possible explanations for the observed differences and to find solutions on how to circumvent these problems. Our data indicate that quantification of Trx1 is more complicated than previously anticipated, which could be related to the biochemical characteristics of Trx1 as a protein, but also to variables related to the capture ELISA method. Firstly, the effect of different multimeric states of Trx1 was investigated. Trx1 can occur as a monomer or form various oligomers under oxidative conditions [2]. Therefore, different sources of rhTrx1 may differ in the content of the various redox forms due to differences in the purification and preparation as well as the condition of their storage. This may have implications on the usage of rhTrx1 as an ELISA standard. To analyse the occurrence of different multimeric states of Trx1 in rhTrx1 standards, rhTrx1 from two sources were analysed by SDS-PAGE. The results showed that rhTrx1 from IMCO contained not only monomeric rhTrx1, but also large fractions of dimers, trimers and multimers. In contrast, rhTrx1 from R&D contained mainly monomeric rhTrx1. Both sources of rhTrx1 were delivered lyophilized, but the R&D rhTrx1 had been treated with DTT, most likely explaining the observed difference.

Consequently, different capture and detection antibody combinations to human Trx1 were tested for differential reactivity in ELISA to the two sources of rhTrx1. Using the mAb for capture and pAb for detection yielded a lower reactivity to monomeric rhTrx1 and higher reactivity to multimeric rhTrx. This implies that an unknown sample with high levels of multimeric Trx1 compared to a standard containing mainly monomeric rhTrx1 would overestimate the level of Trx1 in the sample; and vice versa if a multimeric standard is used to measure a sample with predominantly monomeric Trx1 content, the sample would be underestimated. The pAb/pAb system reacted with both sources of rhTrx, albeit somewhat stronger with multimeric Trx1, indicating that this ELISA system is less sensitive to such misinterpretations. Compared to the mAb/pAb system, the pAb/pAb system quantified higher levels of Trx1 in plasma, which suggests that the redox forms of Trx1 present in plasma are better recognised by this ELISA format. However, both the mAb and pAb used in this study were raised against *E. coli*-derived rhTrx1 and another factor that can affect the quantitation of Trx1 in plasma is how well the antibodies react with endogenous human Trx1 in plasma.

A large number of plasma samples (30–70% depending on the study) contain HA [21] that can interfere with the ELISA analysis by cross-linking the capture and detection antibodies [18], [19], [20], [22], [31]. Two plasma samples with high and low HA content, respectively, were analysed for their content of Trx1. Both mAb and pAb antibody combinations were shown to be affected by HA, but the addition of a HA blocking buffer was able to eliminate the HA interference in both cases. The fact that HA caused false positive results was confirmed by replacing the Trx1-specific capture mAbs with irrelevant antibodies; in the absence of HA blocking buffer, the plasma containing high HA levels yielded similar results compared to the use of Trx1-specific capture antibody.

Notably, only one previous report showing increased levels of Trx1 specifically mentions the possibility of HA interference and the use of a special protocol to remove this activity [16].

To further evaluate the functionality of the ELISA, 18 plasma and 57 CSF samples were analysed by pAb/pAb and mAb/pAb ELISA, respectively, to determine whether these assays detect human Trx1 in the same manner and to test their susceptibility to HA interference. Although Trx1 is known to be a stable protein and may be stored at −20°C for more than a year without degradation, it is regarded as a limitation of the study that the plasma samples were kept at −20°C instead of the recommended −80°C for up to 6 months before use. In the presence of HA blocking buffer the Trx1 levels determined in plasma by the two different ELISAs were in good agreement demonstrating that they react similarly with Trx1 in different plasma. When using HA blocking buffer, the mAb/pAb ELISA could no longer detect Trx1 in 14 of the 57 CSF samples which together with generally lower detection of Trx1 with the mAb/pAb ELISA, compared to the pAb/pAb ELISA, gave rise to a proportional error in the Bland-Altman analysis. However, Spearman’s rank analysis indicated that the correlation between the ELISAs determined in the presence of HA blocking buffer was better (r_s_ = 0.66) compared to not using HA blocking buffer for the analysis (r_s_ = 0.37). As far as we know there is only one earlier study in which Trx1 levels have been determined in CSF and it is unclear if HA were blocked in that study [30], thus the normal range for Trx1 levels in CSF is still unknown. The Trx1 levels determined in CSF in the current study only included CSF from patients and are not representative as normal values. Considering that the two ELISA formats differ in their preferential reactivity with different redox forms of rhTrx1, this may suggest that the redox forms of endogenous Trx1 in plasma do not differ a lot between samples. In contrast, when the CSF samples were analysed without measures taken to prevent HA interference, the levels determined by the two ELISA assays correlated poorly. Not only does this show the problem with HA interference, it also demonstrates that HA affect the two ELISA formats differently. A possible explanation is that the HA affecting the mAb/pAb ELISA have to cross-react with immunoglobulins from several species (in this case mouse and goat) whereas HA with a more limited cross-reactivity between immunoglobulins from different animals cause interference also in the pAb/pAb ELISA.

In conclusion, the present study shows that the design of a Trx1 ELISA with regard to antibodies and standards used have an impact on the measured Trx1 levels. This can explain the varying Trx1 levels in the control groups observed in different studies. Importantly, the analysis of human plasma and CSF without prevention of HA interference may obscure the obtained data. Overall, the results of this study are crucial for the improvement of future studies on the association of Trx1 levels with various diseases.
